# Adverse childhood experiences and mental ill-health - obesity comorbidity among British adolescents – A national cohort study

**DOI:** 10.1177/26335565231215638

**Published:** 2023-11-21

**Authors:** Alexis Karamanos, Amal R. Khanolkar

**Affiliations:** 1 Department of Population Health Sciences, School of Life Course and Population Sciences, 4616 King’s College London, London, UK; 2 Department of Global Public Health, Karolinska Institutet, Stockholm, Sweden

**Keywords:** Adolescent health, obesity, depression, comorbidity, adverse childhood experiences

## Abstract

**Background:**

Mental ill-health and obesity are increasingly prevalent in childhood with both conditions likely to co-occur. Less is known about associations between adverse childhood experiences (ACEs) and mental ill-health and obesity (MH-OB) comorbidity in adolescence. The aim of this study was to examine associations between ACEs and MH-OB comorbidity in adolescents from a national cohort study.

**Methods:**

Participants; 10,734 adolescents (males = 50.3%) from the Millennium Cohort Study with 6 ACEs (for e.g., parental MH, drug/alcohol misuse, physical punishment) collected prospectively between ages 3-11 years. MH-OB comorbidity (binary indicator) was based on objectively measured BMI (for overweight/obesity) and self-reported depression/anxiety at ages 14 and 17. Associations between: 1.total ACE scores (0, 1, 2 or ≥3) and additionally each individual ACE, and MH-OB, were analysed used logistic regression, separately at 14 and 17 years.

**Results:**

At age 14, ACE scores were associated with higher odds for MH-OB comorbidity, with a gradient of increasing odds ratios (OR) with increasing ACEs. Individuals with 1 (OR:1.22[95%CI: 1.1-1.6]), 2 (OR:1.7[1.3-2.3]), or ≥3ACEs (OR:2[1.5-2.6]) had increased odds for MH-OB comorbidity compared to those with 0 ACEs. At age 17, associations between ACE scores and MH-OB were attenuated and observed in individuals with ≥3ACEs (OR:1.54, 1.1-2.3). Parental MH (OR:1.5, 1.2-1.9), intimate-partner violence (OR:1.2, 1.1-1.6), physical punishment (OR:1.3, 1.1-1.6), bullying (OR:2, 1.6-2.5) were associated with MH-OB comorbidity age 14. However, only parental MH (OR:1.5, 1.1-2.1) and bullying (OR:1.6, 1.2-2.1) were associated with MH-OB comorbidity at age 17.

**Conclusion:**

ACEs are associated with increased risk of MH-OB comorbidity in between ages 14 and 17. These findings provide timely opportunity for interventions to reduce risk and are pertinent given that MH and obesity contribute significantly to global burden of disease and track across the lifecourse.

## Introduction

Obesity and mental ill-health contribute significantly to global disease burden.^1-3^ Both conditions are often childhood-onset, have a strong propensity to track across the lifecourse, are chronic and associated with adverse health, social and economic outcomes. Further, they are increasing in prevalence in adolescence with evidence for earlier onset in childhood in recent generations.^4^ Globally, the number of children and adolescents (5-19 years old) with obesity has increased 10-fold (11 to 124 million between 1975-2016).^5^ Internalised mental health like depression and anxiety are the most common mental health (MH) disorders, affecting >12% of adolescents and is one of the main causes of disability globally.^6^

Substantial evidence shows the co-occurrence (or comorbidity) of mental ill-health and obesity (MH-OB) in children and adults.^7-11^ Obesity may precede depression as people with obesity are at greater risk of depression due to physical inactivity, poorer quality of life, social discrimination and poor self-esteem.^12^ A reverse temporal sequence is also possible with depression preceding poor lifestyle changes and the use of antidepressant medications, resulting in weight gain. Shared genetics, health behaviours and socioeconomic disadvantage can contribute to the co-occurrence of obesity and mental ill-health.^13,14^ Evidence for co-occurrence of obesity and mental ill-health in adolescence is more limited with most studies being cross-sectional in design but studies indicate that both conditions may track together across childhood.^11,15^

A substantial body of evidence indicates that adverse experiences in childhood (for example, individuals experiencing parental mental ill-health and familial hardships, emotional, sexual and physical abuse, neglect etc.) are associated with a range of adverse health and poorer social outcomes, and health-related behaviours in adolescence and across the life course.^16,17^ While there is less consensus on the definition, measurement and timing of adverse childhood experiences (ACEs), their short- and long-term deleterious impact on health are increasingly being recognised as a public health concern. Prevalence of ACEs in the general population are substantially high (with estimates ranging from 20.1 to 50% for 1 ACE and >24% for ≥2ACEs in adults in England).^18^ Short-term consequences (i.e., in adolescence) of ACEs include obesity, depression, substance misuse, unemployment, less likely to enrol in higher education and violent offending.^19,20^ Further, the magnitude of associations depend on the type, timing and combination of ACEs being used in studies.^21^ While the impact of multiple ACEs on health have been identified, the deleterious impact of specific individual ACEs has also been highlighted. For instance, considerable evidence suggests associations between bullying and a rise in childhood obesity over time. Moreover, obese children are more likely to be bullied due to their weight status, which can subsequently lead to further increase in adiposity.^22^ These observations are corroborated by the diathesis-stress model, which posits that the stress resulting from bullying, combined with biological vulnerability or diathesis, can trigger emotional eating and decrease physical activity, thereby potentially inducing weight gain.^23^

Other factors can also influence associations between ACEs and MH and obesity, including socioeconomic circumstances and the presence of peer and family support. Children from socioeconomically disadvantaged backgrounds are more likely to experience ACEs and have higher risk for a range of health outcomes including obesity and mental ill-health.^24^ Consequently, the escalating rates of childhood obesity in the UK, particularly among the most disadvantaged children, are not a random occurrence. Studies reveal that children from the most deprived backgrounds (31.3%) are over twice as likely to be afflicted with obesity compared to peers from the least deprived (13.5%).^25^ Further, mental ill-health like depression and anxiety have also increased in adolescents in recent years, further exacerbated by the recent Covid-19 pandemic (22). Further, adolescents identifying as sexual minority are at higher risk for some ACEs including bullying and sexual abuse.^26^ Some evidence indicates that children of different ethnic groups experience different rates, types and number of ACEs. For example, in the US, Black and Hispanic children were more likely to experience certain types of ACEs and have a higher number of total ACEs.^27^ It is thus imperative to examine the role of socioeconomic circumstances, sexual identity and ethnicity in the associations between ACEs and MH-OB comorbidity.

Public health policies and interventions have had limited success in tackling obesity and mental health in childhood as both conditions are complex, multifactorial with interrelated and interdependent factors.^23,24^ One possible reason could be the need to understand and tackle co-occurring conditions together. Thus, intervention programmes need to be more comprehensive including identifying new risk factors like ACEs for both conditions including commonalities. This can provide explanation for existing and persistent inequalities in mental ill-health and obesity morbidity in adolescence and across the lifecourse.

We hypothesised that children with ACEs are more likely to have MH-OB comorbidity compared to those without. Further we hypothesised that ethnic-, sexual-minority and/or socioeconomically disadvantaged children with ACEs would be at even greater risk for comorbidity compared to their heterosexual, White and socioeconomically advantaged peers.

The aim of this study was to examine whether exposure to ACEs (combined and individual) between ages 3 and 11 years are associated with increased risk for comorbidity in later adolescence (ages 14 and 17 years), in a nationally representative sample of children. Further, we examined whether associations between ACEs and comorbidity differed by ethnicity, sexual identity and parental socioeconomic position.

## Methods

### Study design and participants

This longitudinal study uses data from the Millennium Cohort Study (MCS), a nationally representative birth cohort following children born Sept 2000-Jan 2002.^28^ A national sample of 19,517 children from across the UK were recruited to MCS and followed-up over seven sweeps (ages 9 months, 3, 5, 7, 11, 14 and 17 years). MCS was oversampled to have higher proportions of ethnic minority (EM) participants and socioeconomically disadvantaged families (with appropriate sample weighting ensuring national representativeness). Attrition across follow-up was predicted by single-parent families, lower-income occupation and lower educational level, Black ethnicity, and male sex. This study includes N = 10,734 children at age 14 and N = 9,331 children at age 17 having data on any ACE from the ages 3, 5, 7 and 11 sweeps *and* mental health and BMI from ages 14 or 17.

Ethics approval for the MCS study was obtained from the National Research Ethics Service Committee London— Central (reference 13/LO/1786). All data are anonymized and available to researchers via the UK Data Service. Cohort members ≥16 years provided verbal consent to take part in the overall assessment, and each survey element.

### Exposures of interest

Information on six ACEs (parental problem drinking behaviour, drug use, maternal psychological distress, intimate partner violence, physical punishment of children and bullying) were obtained from age 3 (MCS sweep 2) through to age 11 (MCS sweep 5) and based on self-report of MCS cohort members or their main carers (in most cases their mother). We included information on five ACEs from multiple questions to main carers to generate binary variables for each of the following: regular maternal drug use, maternal alcohol problem drinking, parental psychological distress, intimate partner violence, and physical punishment. The sixth – experiences of bullying – was answered by cohort members. Supplemental Table 1 provides complete details on the ACEs and outcomes of interest.

Based on the above binary ACE variables, we created a cumulative adversity score (hereafter ACE score) indicating the following categories: 0 ACEs, 1 ACE, 2 ACEs and ≥3ACEs.

### Mental ill-health and overweight/obesity comorbidity at ages 14 and 17

#### Mental health

The Short Moods and Feelings Questionnaire (SMFQ), a 13-item (for e.g., I felt miserable or unhappy) self-report measure of depressive symptoms was used to assess MH at age 14.^29^ Response to items are scored and summed to give an overall score ranging from 0 to 26 (higher scores indicating presence of depressive symptoms). Total scores were categorised into a binary variable based on recommended cut-off points (≥8 indicating depressive symptoms).^30^ We used two indicators of mental health at age 17: the emotional symptoms subscale of the Strengths and Difficulties Questionnaire (SDQ-S) and additionally the 6-item Kessler Psychological Distress Scale (K6).^31,32^

The SDQ-S emotional symptoms subscale comprises 5 items (for e.g., are you often unhappy?) which assess symptoms of psychological distress in the past six months. Participants could respond with: Not true, somewhat true, or certainly true. Total scores were calculated with a minimum zero score indicating no problems to a maximum possible score of 10, with higher scores indicating more emotional problems (like depression and anxiety). Total scores were categorised into a binary variable based on recommended cut-off points indicating participants with ‘close to average’ (<6) vs. ‘high/very high levels’ (≥6) of difficulties.^33^

The K6 assesses symptoms of nonspecific psychological distress (for example, how often do you feel depressed?) in the preceding 4 weeks. Respondents chose one of five answers (none of the time, a little of the time, some of the time, most of the time or all the time). Items were scored to create an overall score ranging from 0 to 24 (higher scores indicate greater levels of depression and anxiety symptoms). The total score was categorised into a binary variable (scores ≥13 indicating nonspecific serious psychological distress or diagnosable mental illness).^34^

### Overweight and obesity

Cohort members’ heights were measured at home using a Leicester height measure stadiometer. Weight measurements were taken using Tanita scales (BF–522W). All interviewers were trained in using this equipment (Fitzsimons et al., 2020). BMI was calculated as weight divided by height(squared). Participants were classified as normal BMI or obese (including overweight) using the International Obesity Task Force age- and sex-specific cut-offs for 2-18-year-olds.^35^ Participants who were underweight were very small in number and included in the normal BMI category.

### Mental ill-health and overweight/obesity comorbidity

We created a binary variable which indicated those individuals without and with comorbidity. Two comorbidity indicators were created using either indicator of MH at age 17. Individuals who only had either mental ill-health or overweight/obesity were coded as having no comorbidity.

### Other covariates

Parental income was used as an indicator of socioeconomic position ascertained at age 3. We chose to use parental income measured at age 3 as socioeconomic position in early childhood is associated with higher risk for ACEs (which are measured from age 3 onward). Household income (Organisation for Economic Co-operation and Development UK) was categorized into equalized quintiles (where quintiles 1 and 5 represent the lowest and highest income quintiles respectively). Parent/guardian reported participant’s ethnicity at age 3 (Supplementary Table 1). Reported ethnicity at subsequent sweeps was used if information was not available at age 3. For analysis, subjects were grouped into either 1. White (ethnic majority and reference category), 2. Non-White (mixed ethnicity, South Asian, Black or ‘other’). The ‘other’ group included participants from Asia (excluding South Asia), the Middle East and South America. Sex was based on sex at birth. Based on self-reported answers on sexual identity (from eight options listed in Supplementary Table 1), participants were categorized into 1. Completely heterosexual (reference category) and 2. Sexual minority (mainly heterosexual/straight, bisexual, mainly gay/lesbian, and completely gay/lesbian). The original questions, complete component items of each scale and all health- and ACE-related indicators, and how they were categorized (including references) are explained in detail in Supplementary Table 1.

### Missing data

Due to attrition over time in MCS, significant data on ACEs was missing (ranging from 3.2% for bullying to 58% for paternal depressive symptoms). With respect to the covariates, data was largely complete, although sexual identity had a higher proportion (16.4%) of missing data. Missing data was addressed using Multiple Imputation by Chained Equations (MICE), operating under the Missing at Random Assumption (MAR). This was suitable given that observed characteristics like ethnicity and parental income were independent predictors of missingness (i.e., ethnic minority and socioeconomically disadvantaged participants were more likely to have missing information across ≥1 analysis parameters). In order to predict missing data, the imputation model integrated all ACEs and covariates, and auxiliary variables like maternal age at birth and parental highest educational qualifications. We also included interaction terms between individual ACEs, sex, ethnicity, family income, and sexual identity. Given that missing data for all analysis parameters amounted to 80% or more, we generated 80 multiple imputations. We then combined these estimates using Rubin's rules. To guard against potentially problematic imputations, we discarded imputed values on comorbidity at ages 14 and 17 during multivariable modelling.^36^

Our comparison of characteristics, using complete cases and the multiple imputed data (as displayed in Supplementary Table 2), confirmed that the use of multiple imputation was appropriate. Further, comparison of the results obtained from the complete cases and imputed analysis showed that the standard errors were smaller for multiple imputation, demonstrating the efficiency gain of multiple imputation over complete cases analysis. This helped to avoid bias in the resulting estimates, which could have been attributed to the disproportionate contribution of more socio-economically advantaged participants (which could lead to underestimation of ACE-outcome associations).

### Statistical analysis

We used logistic regression models to assess associations between 1. Each individual ACE and 2. Cumulative ACE scores and comorbidity separately at ages 14 and 17. We first ran models to assess crude associations followed by multivariable models adjusted for sex, sexual identity, ethnicity and parental income.

*Interactions* – We also assessed whether associations between cumulative ACE scores and comorbidity differed by ethnicity, sexual identity or parental income. The above multivariable logistic regression models were run with interaction terms between cumulative ACE scores and ethnicity, parental income or sexual identity (with adjustment for covariates).

All models were weighted with non-response weights from the birth sweep to account for the stratified cluster design of the MCS and attrition over time (using the Stata ‘*svy*’ command for survey data). Odds ratios from models were plotted for visualisation. Analyses were conducted in Stata V17 (StataCorp LP, College Station, Texas).

### Sensitivity analysis

We also ran the above multivariable logistic regression models using total ACE scores as defined previously but excluding parental psychological distress and bullying. For this analysis, total ACE scores were categorized as 0, 1, and ≥2 (due to the fewer number of ACEs). The goal of this adjustment was to examine whether the associations between total ACE scores and comorbidity were primarily driven by parental psychological distress and bullying.

## Results

Descriptive statistics are reported in Table 1 and Supplemental Table 2. Between ages 3 and 11 years, 44.3% of participants did not experience any ACE, whereas 29.4, 14.7 and 11.5% experienced 1, 2 and ≥3ACEs, respectively. The two most common ACEs were physical punishment (28.5%) and maternal psychological distress (25.8%). 25% of the sample was either overweight or obese at age 14 increasing to 30.1% at age 17. 15.9% had symptoms of psychological distress at age 14 which increased to 22.2% at age 17. The prevalence of comorbidity progressively increased with the number of ACEs (for example at age 14, 6.4% of individuals with 0 ACEs had comorbidity which increased to 11.9% for those with ≥3ACEs). Individuals exposed to maternal psychological distress, parental intimate partner violence and bullying were more likely to have comorbidity at age 14.

**Table 1.** Descriptive characteristics of 10,734 adolescents by mental ill-health and overweight or obesity comorbidity status that attended the ages 14- and 17-years assessments of the Millennium Cohort Study. All Values are %.

Characteristic	Comorbidity at age 14 (N = 10,734)				Comorbidity at age 17 (N = 9,936)-Kessler^1^				Comorbidity at age 17 (N = 9,930)-SDQ^2^			
Cumulative adverse childhood experiences (ACEs) score	%	95% CI		*p*	%	95% CI		*p*	%	95% CI		*p*
0 ACE	6.4	5.5	7.3	<0.001	3.8	3.1	4.4	<0.001	6.2	5.3	7.0	0.141
1 ACE	7.7	6.5	9.0		5.1	4.1	6.1		7.0	5.9	8.1	
2 ACEs	10.7	8.6	12.9		6.4	4.9	7.9		7.6	6.0	9.3	
≥3 ACEs	11.9	9.6	14.3		7.1	5.4	8.8		7.7	5.9	9.5	
Individual ACEs												
Regular maternal drug use No	7.9	7.4	8.6	0.39	5.0	4.6	5.5	0.57	6.8	6.3	7.3	0.168
Yes	10.4	4.4	16.4		4.2	0.0	8.4		10.9	4.7	17.0	
Maternal problem drinking behaviour												
No	7.9	7.4	8.6	0.89	5.0	4.5	5.5	0.78	6.8	6.2	7.3	0.844
Yes	8.2	5.7	10.7		5.5	3.5	7.5		7.5	5.2	9.8	
Maternal psychological distress												
No	7.0	6.3	7.8	<0.001	4.1	3.5	4.6	<0.001	6.3	5.6	7.0	0.025
Yes	11.1	9.6	12.7		7.3	6.2	8.4		8.1	6.9	9.4	
Bullying												
No	7.1	6.5	7.7	<0.001	4.3	3.8	4.8	<0.001	6.3	5.8	6.9	<0.001
Yes	13.5	11.5	15.5		9.0	7.4	10.5		9.8	8.1	11.4	
Physical punishment												
No	7.7	6.9	8.4	0.157	5.1	4.6	5.7	0.660	7.3	6.6	7.9	0.426
Yes	8.8	7.6	10.0		4.8	3.9	5.6		5.9	5.0	6.8	
Intimate Partner Violence												
No	7.6	6.9	8.3	<0.05	4.8	4.2	5.3	0.125	6.7	6.1	7.4	0.738
Yes	9.9	8.2	11.7		6.0	4.6	7.4		7.3	5.8	8.7	
Mental ill-heath and overweight or obesity	8.0	7.4	8.6		4.7	4.1	5.3		6.8	6.1	7.4	
Sex at birth												
Male	4.9	4.3	5.6	<0.001	2.6	2.1	3.2	<0.001	3.4	2.8	4.1	<0.001
Female	11.2	10.3	12.1		6.8	6.0	7.7		9.9	8.9	10.9	
Ethnicity												
White	8.1	7.4	8.7	<0.05	4.8	4.2	5.4	0.002	6.9	6.3	7.6	0.007
Mixed ethnicity	10.1	6.1	14.2		6.3	2.9	9.8		8.1	3.9	12.3	
South Asian	5.3	3.6	7.1		3.3	2.0	4.5		3.5	2.2	4.7	
Black	11.7	7.2	16.3		5.9	2.6	9.2		4.8	1.7	7.9	
Other ethnic minority	6.9	2.3	11.5		2.8	0.5	5.0		5.5	0.4	10.6	
Sexual identity												
Exclusively heterosexual	6.5	5.9	7.1	<0.001	2.9	2.4	3.3	<0.001	4.7	4.1	5.3	<0.001
Sexual minority	13.5	11.7	15.4		11.3	9.3	13.2		13.7	11.7	15.7	
Equivalised household income												
Bottom quintile	10.5	9.0	12.1	<0.001	6.5	5.4	7.7	<0.001	7.6	6.4	8.9	<0.001
40%	10.4	8.8	11.9		6.2	5.1	7.3		8.5	7.2	9.8	
60%	7.9	6.5	9.1		5.1	4.1	6.1		7.0	5.9	8.2	
80%	7.5	6.2	8.8		4.3	3.4	5.2		6.2	5.1	7.3	
Highest quintile	5.4	4.3	6.6		3.1	2.3	3.8		4.9	4.0	5.9	

1 Mental health component based on 6-item Kessler Psychological Distress Scale (K6).

2 Mental health component based on Strengths and Difficulties Questionnaire (SDQ-S) emotional symptoms subscale.

### Associations between individual ACEs and comorbidity

**Figure 1.**
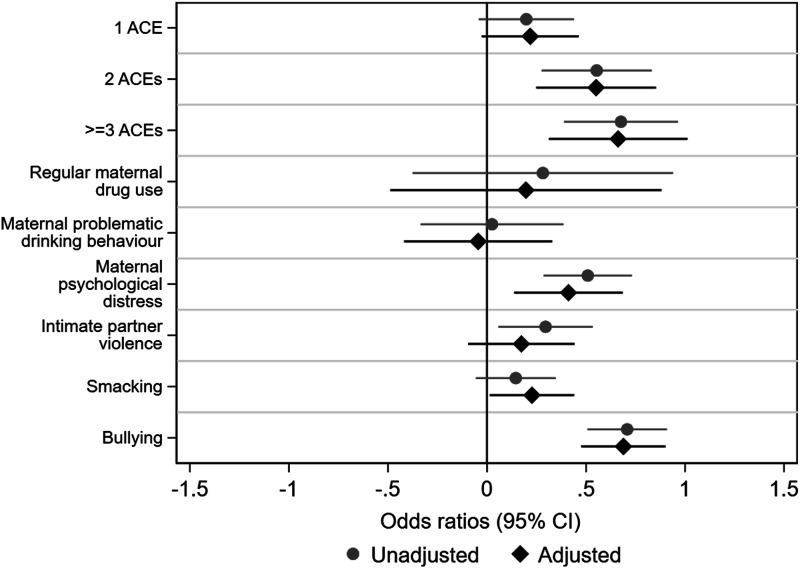
Odds ratios for mental ill-health and obesity comorbidity in relation to adverse childhood experiences (ACEs) at age 14 in 10,734 adolescents from the Millennium Cohort Study. Note: X-axis is on the logarithmic scale.

**Figure 2.**
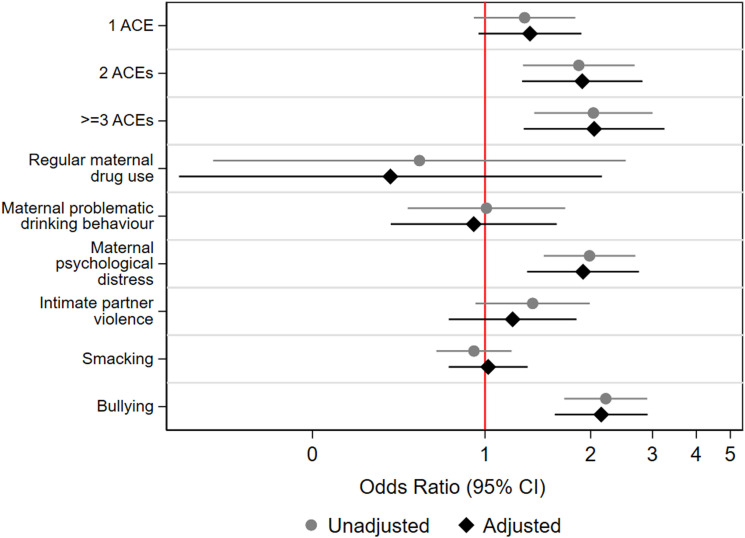
Odds ratios for mental ill-health and obesity comorbidity in relation to adverse childhood experiences (ACEs) at age 17 in 9,331 adolescents from the Millennium Cohort Study. Mental health based on the on 6-item Kessler Psychological Distress Scale (K6).

**Figure 3.**
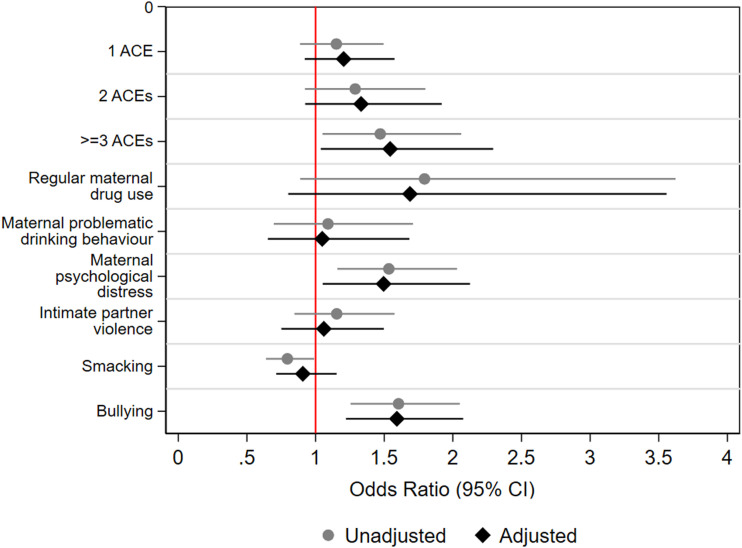
Odds ratios for mental ill-health and obesity comorbidity in relation to adverse childhood experiences (ACEs) at age 17 in 9,331 adolescents from the Millennium Cohort Study. Mental health based on the Strengths and Difficulties Questionnaire (SDQ-S) emotional symptoms subscale.

Supplemental Tables 3 and 4, and Figures 1, 2, 3, display results from logistic regression models examining associations between individual ACEs and comorbidity at ages 14 and 17, respectively. At age 14, we found that maternal psychological distress (OR 1.66, 95% CI: 1.33-2.08), intimate partner violence (OR 1.35, 1.10-2.08) and bullying (OR 2.01, 1.66-2.48) were significantly associated with increased odds for comorbidity. Adjusting for confounders marginally attenuated these observed odd ratios. The remaining ACEs (maternal drug use, problem drinking behaviour, and physical punishment) were also associated with increased odds for comorbidity but 95% confidence intervals included 1. However, physical punishment was found to be significantly associated with increased odds (OR 1.26, 1.01-1.55) for comorbidity after adjustment for confounders.

We found limited evidence for associations between individual ACEs and comorbidity at age 17. Only parental psychological distress (OR 1.98, 1.47-2.68) and bullying (OR 2.21, 1.68-2.90) were associated with increased risk for comorbidity.

### Associations between cumulative ACE scores and comorbidity

Supplemental Tables 3 and 4, and Figures 1, 2, 3 display results from logistic regression models examining associations between cumulative ACE scores and comorbidity at ages 14 and 17, respectively. At both ages, cumulative ACE scores were associated with increased odds for comorbidity (with stronger and more consistent associations at age 14 compared to age 17). Further, at both ages, we observed a significant gradient: increasing number of ACEs were progressively associated with increasing odds for comorbidity (for example, at age 14, individuals with 1, 2 or ≥3ACEs had OR 1.22 [1.01-1.55], 1.74 [1.31-2.29] and 2.00 [1.48-2.62], respectively, higher odds for comorbidity compared to those with no ACEs, test for trend; p < 0.01). However, at age 17, we observed that the 95% CIs for OR for 1 ACE (in case of K-6) and OR for 1 ACE and 2 ACEs (in case of SDQ) included 1.

We observed more consistent and stronger associations between cumulative ACE scores and comorbidity with K-6 as the indicator of mental health, compared to SDQ (for example, with K-6, ≥3ACEs: OR 2.05 [1.29-3.25] and with SDQ, ≥3ACEs: OR 1.54 [1.04-2.29]).

Multivariable logistic regression models including interaction terms between cumulative ACE scores and MH-OB comorbidity (to assess whether associations differed by ethnicity, sexual identity or parental income) were not significant (Supplementary Tables 6 and 7).

### Sensitivity analysis

Associations between total ACE scores (excluding maternal psychological distress and bullying) and comorbidity were substantially attenuated at both ages (Supplemental Table 5). In these models, individuals with 2 ACEs had higher odds for comorbidity [(OR 1.73, 1.22-2.45) at age 14, but not at age at age 17], compared to those with no ACEs.

## Discussion

In our study, comprising 10,734 adolescents from a national cohort, ACEs occurring early in life (between ages 3 to 11) were associated with a higher likelihood of mental health problems and obesity comorbidity in later adolescence (ages 14 and 17). This association was evident with certain individual ACEs (like maternal psychological distress) and higher cumulative ACE scores.

Notably, associations between ACEs and comorbidity were more robust at age 14 than at age 17. Among various ACEs examined, bullying and maternal psychological distress exhibited the most consistent and significant associations with adolescent comorbidity. Familial socioeconomic circumstances, ethnicity, and sexual identity did not significantly affect the relationship between ACEs and comorbidity.

In our examination of cumulative ACE scores excluding maternal psychological distress and bullying, we found that a composite score, which included maternal drug use, physical punishment, problematic alcohol drinking behaviour, and domestic abuse was associated with a considerably higher likelihood of comorbidity at age 14 but not age 17. This observation suggests that the cumulative impact of these specific ACEs might be more substantial at younger ages during childhood.

### Strengths and weaknesses

Significant strengths include a large sample size with rich social, economic and health data drawn from a well-studied nationally representative birth cohort facilitating generalisability to the UK adolescent population. The use of prospectively collected ACE data reduces possibilities of recall bias and reverse causation. Further, using commonly studied ACEs (and operationalised as total scores and individual entities) aids comparisons with existing studies. The MH-OB comorbidity outcome was separately estimated using two widely validated indicators of mental health. Finding similar associations with both versions of the outcome ensure consistent findings. Another strength is the use of objectively measured BMI to compute overweight/obesity status.

Like most longitudinal studies, MCS suffers from attrition (of the original 19,517 children, 10,757 attended the age 17 sweep) but this was addressed using the robust multiple imputation technique. While imputation relies on the MAR assumption which is not empirically verifiable,^36^ we increased the plausibility of the MAR assumption by including a rich set of auxiliary variables (maternal age at birth, parental education and vicarious racism at age 5) in the imputation model. Auxiliary variables help increase the precision of predicting missing data and minimising non-random variation in values. Additionally, longitudinal data on ACEs, BMI, and mental health and high-quality socioeconomic measures are powerful predictors of missing data which significantly strengthen the imputation model.

We used two measurements of ACEs; total ACE score and individual ACEs, and both operationalisations have their strengths and weaknesses. Using total ACE scores assumes that each constituent ACE is equally important for outcomes which is unlikely. But this approach facilitates comparisons with similar studies as total ACE scores are commonly used. We also examined each ACE independently as substantial evidence shows that certain ACEs like bullying and parental inter-partner violence can be more detrimental for offspring health. Further, examining individual ACEs gives some indication of those that might be more detrimental to health in later adolescence and can be prioritised for targeted public health intervention. A limitation of note is that we were unable to include relevant ACEs like sexual abuse, ethnic - and sexual-identity related abuse (like racism and bullying) due to paucity of such prospectively collected data earlier in childhood. Further, as mothers were respondents for the majority of children (approximately 95%), we were unable to independently examine impact of paternal mental health and health-risk behaviours as ACEs and adversities experienced by parents outside the family as these were uncaptured. Another limitation is the measurement bias associated with some ACEs. For example, parental alcohol misuse should be treated as an under-estimation due to possible reporting bias. Moreover, only some of the items were available for measuring parental discord and harsh parenting in the original GRIMS scale. Reporting bias might exist for some of the parent-reported ACEs as some parents might be less inclined to reveal certain sensitive information like drug and alcohol misuse, potentially resulting in misclassification and underestimation of some ACEs. We did not find any evidence for interactions with sex, family income, ethnic- and sexual-identities but this could be because of lack of statistical power. Finally, due to the observational nature of our study, we cannot eliminate the possibility of unmeasured confounding. Consequently, the associations we examined and the results we obtained should not be interpreted as establishing causal inferences.

### Comparison with existing literature

To our knowledge no study has examined associations between ACEs and mental ill-health and obesity comorbidity in adolescence. Several studies (including those on the MCS) have separately examined associations between ACEs and mental health, and to a lesser extent ACEs and obesity in adulthood. Evidence for associations between ACEs and indicators of adiposity (including BMI and obesity) in adolescence are more limited. A previous longitudinal study on participants from the MCS found that boys who experienced ≥3ACEs, and both boys and girls who experienced parental depression had steeper BMI trajectories in adolescence.^37^ Another study with participants from Brazil and the UK found limited evidence for associations between ACEs and adiposity in UK adolescents but not in Brazilian counterparts.^38^ One explanation could be a lag time effect wherein the impact of ACEs on obesity is seen later in adulthood.^39^ However, the evidence on associations between ACEs and mental health especially depression is much stronger. A longitudinal study on MCS found that ACEs were associated with worse mental health across childhood and adolescence with a clear dose-response relationship.^20^ This finding has been shown by several other studies, both in relation to cumulative ACE scores and individual ACEs like parental depression, discord and harsh parenting.^40,41^ A study on another comparable UK cohort of adolescents found that adjustment for socioeconomic indicators only marginally attenuated ACE – depression associations and limited evidence for effect modification, in line with our findings.^40^ A recent meta-analysis comprising >133,000 adolescents showed that bullying was associated with nearly 3 times the risk of depression compared to those not bullied.^42^ Further, overweight/obese individuals experienced significantly higher risk to be bullied.^43^ This provides for a ‘negative feedback loop’ wherein which bullying may lead to subsequent depression and consequent weight-gain which then results in these individuals be further bullied.

The proportion of adolescents living with both obesity and depression is much smaller than those having either condition alone as observed in our study sample (7.1% had comorbidity compared to 30% with obesity and 22.2% with depression at age 17). This highlights a selective group of individuals living with comorbidity and often overlooked as most studies examine the impact of ACEs on individual health and social outcomes.

### Implications, policy, future research

Our findings clearly show that ACEs have deleterious associations with mental ill-health and obesity comorbidity in later adolescence. However, the large body of evidence showing associations between several ACEs and health both substantially strong in effect size and with clear gradients replicated in multiple populations across countries indicate that these associations are more likely causal. Nonetheless, our findings indicate that primary prevention or minimising ACEs is crucial. While it is not realistic to prevent all ACEs from occurring, the deleterious impact of ACEs needs to be highlighted in wider society raising awareness, which may reduce the prevalence of some ACEs. However, effective policies can be developed to tackle certain ACEs with prevention as a goal. These include bullying in schools and elsewhere. Further, effective policies need to be developed to 1. Reduce the impact of some ACEs such as providing timely intervention and care for poor mental health and individuals with health-risk behaviours among both parents and their children. 2. Increasing awareness among healthcare service providers about both the adverse consequences of ACEs on individuals in general, and more specifically how it is associated with comorbidities. This is pertinent as living with mental ill-health and obesity comorbidity is also linked to increased risk for other health conditions. Further, policies on treatment can be tailored according to the number and type of ACEs for different demographic groups as our findings suggest both increased risk for adverse health in relation to the number of ACEs and the type of ACEs.

There is substantial scope for future research. Our findings need to be replicated in comparable adolescents in other countries and settings and could include ACEs we lacked information on. Whether the higher likelihood for comorbidity in relation to ACEs persists into adulthood is unexplored. While we did not find evidence for effect modification by sexual- and/or ethnic identity and socioeconomic position, this needs further investigation in larger samples with adequate representation of minority groups. Lack of evidence on effect modification by ethnicity, sexual identity and socioeconomic position does not indicate that there are no variations. Perhaps it does not exist in these data or we were unable to capture it in the MCS due to lack of statistical power. Nonetheless, this is particularly important to investigate as relatively little is known about how ACEs impact health in ethnic minority youth and individuals living with multiple minority identities (for example, sexual-, ethnic- and religious-minority identities). Future studies should also include other ACEs not examined here as well as other operationalisations of ACEs. Our data relates to pre-pandemic years (before March 2019), and studies need to examine whether the impact of ACEs on comorbidity has increased recently.

### Final summary

Our study found that exposure to cumulative ACEs in early childhood increased the likelihood for MH-OB comorbidity at age 14 but not age 17. Nonetheless, certain ACEs like maternal psychological distress and bullying were consistently associated with comorbidity at both ages 14 and 17. There is considerable scope to further examine these relationships in comparable populations but there is also a clear need for greater and more effective public health intervention to reduce the impact of ACEs on health.

## Supplemental Material

Supplemental Material - Adverse childhood experiences and mental ill-health - obesity comorbidity among British adolescents – A national cohort study Click here for additional data file. Supplemental Material for Adverse childhood experiences and mental ill-health - obesity comorbidity among British adolescents – A national cohort study by Alexis Karamanos and Amal R. Khanolkar in Journal of Multimorbidity and Comorbidity
