# Supplementary material for: Adverse childhood experiences and mental ill-health - obesity comorbidity among British adolescents – A national cohort study
Source: J Multimorb Comorb. 2023 Nov 21;13:26335565231215638. doi: 10.1177/26335565231215638 (PMC10664438; doi:10.1177/26335565231215638)
Supplement: Supplemental Material - Adverse childhood experiences and mental ill-health - obesity comorbidity among British adolescents – A national cohort study [file sj-pdf-1-cob-10.1177_26335565231215638.pdf]

# *Supplemental Data*

**Adverse childhood experiences and mental health-obesity comorbidity among British adolescents – A national cohort study**

**Alexis Karamanos[1] and Amal R. Khanolkar[1,2]**

1. Department of Population Health Sciences, School of Life Course and Population Sciences, King's College London, London, UK. 2. Department of Global Public Health, Karolinska Institutet, Stockholm, Sweden

**Supplemental Table 1. A detailed description of adverse childhood experiences and mental health indicators recorded in the Millennium Cohort Study and used in this study**

| | Reported | Available observations | Criterium/Dichotomisation | MCS Sweep | Age in years |
|---|---|---|---|---|---|
| *Adverse childhood experiences (ACEs)* | | | | | |
| **Problem drinking behaviour** | Mother | 7,854 | CAGE questionnaire- a score of 2 and above indicating problem drinking behaviour | MCS 2 | Age 3 |
| **Problem drinking behaviour** | Mother | 7,216 | Positive responses to all 3 questions; Not being able stop drinking, failed to do as expected due to drinking, relatives and friends have been concerned about drinking behaviour | MCS 5 | Age 11 |
| **Maternal problem drinking behaviour** | Mother | 6,093 | Problem Behaviour Drinking at MCS 2 or MCS 5 | | |
| **Use of drugs the past 12 months** | Mother | 9,355 | 0 = Never/occasionally, 1 = Regularly | MCS 2 | Age 3 |
| **Use of drugs the past 12 months** | Mother | 10,368 | 0 = Never/occasionally, 1 = Regularly | MCS 3 | Age 5 |
| **Regular maternal drug use** | Mother | 8,721 | Use of drugs at MCS 2 or MCS 3 or MCS 6 | | |
| **Maternal psychological distress** | Mother | 8,762 | Kessler scale, 0 = score 0/12, 1 = 13/max | MCS 2 | Age 3 |
| **Maternal psychological distress** | Mother | 10,523 | Kessler scale, 0 = score 0/12, 1 = 13/max | MCS 3 | Age 5 |
| **Maternal psychological distress** | Mother | 10,371 | Kessler scale, 0 = score 0/12, 1 = 13/max | MCS 4 | Age 7 |
| **Maternal psychological distress** | Mother | 9,971 | Kessler scale, 0 = score 0/12, 1 = 13/max | MCS 5 | Age 11 |
| **Maternal psychological distress** | Mother | 7,656 | Score of 13 or above at MCS 2 or MCS 3 or MCS 4 or MCS 5 | | |
| **Paternal psychological distress** | Father | 6,977 | Kessler scale, 0 = score 0/12, 1 = 13/max | MCS 2 | Age 3 |
| **Paternal psychological distress** | Father | 7,758 | Kessler scale, 0 = score 0/12, 1 = 13/max | MCS 3 | Age 5 |
| **Paternal psychological distress** | Father | 7,362 | Kessler scale, 0 = score 0/12, 1 = 13/max | MCS 4 | Age 7 |
| **Paternal psychological distress** | Father | 6,395 | Kessler scale, 0 = score 0/12, 1 = 13/max | MCS 5 | Age 11 |
| **Paternal psychological distress** | Father | 4,436 | Score of 13 or above at MCS 2 or MCS 3 or MCS 4 or MCS 5 | | |
| **Parental psychological distress** | Mother/Father | 3,697 | Score of 13 or above at MCS 2 or MCS 3 or MCS 4 or MCS 5 | | |
| **Physical punishment** | Mother | 9,325 | 0 = Never/rarely, 1 = Once a month, once a week or more often, daily | MCS 2 | Age 3 |
| **Physical punishment** | Mother | 10,465 | 0 = Never/rarely, 1 = Once a month, once a week or more often, daily | MCS 3 | Age 5 |

| | | | | | |
|---|---|---|---|---|---|
| **Physical punishment** | Mother | 10,326 | 0 = Never/rarely, 1 = Once a month, once a week or more often, daily | MCS 4 | Age 7 |
| **Physical punishment** | Mother | 8,555 | Child was physically punished at MCS 2 or MCS 3 or MCS 4 | | |
| **Partner has ever used force in the relationship?** | Mother | 7,824 | No vs Yes | MCS 2 | Age 3 |
| **Partner has ever used force in the relationship?** | Mother | 8,477 | No vs Yes | MCS 3 | Age 5 |
| **Partner has ever used force in the relationship?** | Mother | 8,208 | No vs Yes | MCS 4 | Age 7 |
| **Partner has ever used force in the relationship?** | Mother | 7,956 | No vs Yes | MCS 5 | Age 11 |
| **Intimate partner violence** | Mother | 6,196 | Positive answers at MCS2 or MCS3 or MCS4 or MCS5 or MCS6 | | |
| **How often do other children bully you?** | Child | 10,161 | 0=Never/Some of the time, 1=All the time | MCS 4 | Age 7 |
| **How often other children hurt you or pick on you on purpose?** | Child | 10,885 | 0 = Never/about once a month, 1 = About once a week/most days | MCS 5 | Age 11 |
| **Bullying** | Child | 9,724 | Bullied at MCS 4 or MCS 5 | | |
| ***Mental health indicators in adolescence*** | *Component questions* | | | | |
| **Short Moods and Feelings Questionnaire (SMFQ)** | I felt miserable or unhappy<br>I didn't enjoy anything at all<br>I felt so tired, I just sat around and did nothing<br>I was very restless<br>I felt I was no good anymore<br>I cried a lot<br>I found it hard to think properly or concentrate<br>I hated myself<br>I was a bad person<br>I felt lonely<br>I thought nobody loved me<br>I thought I could never be as good as other kids<br>I did everything wrong | | *Options:*<br>1. Not true<br>2. Sometimes true<br>3. True<br><br>Form used in analysis: binary | MCS 6 | Age 14 |
| **Self-reported Strengths and Difficulties Questionnaire (SDQ)** *-emotional symptoms subscale* | -Complains of headaches/stomach aches/sickness<br>-Often seems worried<br>-Often unhappy<br>-Nervous or clingy in new situations<br>-Many fears, easily scared. | | *Options:*<br>1. Not true<br>2. Somewhat true<br>3. Certainly true<br><br>Form used in analysis: binary | MCS7 | Age 17 |
| **Self-reported Kessler Psychological Distress Scale (6 item)** | During the last 30 days about how often:<br>- did you feel so depressed that nothing could cheer you up?<br>- did you feel hopeless?<br>- did you feel restless or fidgety?<br>- did you feel that everything was an effort?<br>- did you feel worthless?<br>- did you feel nervous? | | *Options:*<br>1. All of the time<br>2. Most of the time<br>3. Some of the time<br>4. A little of the time<br>5. None of the time<br><br>Form used in analysis: binary | MCS 7 | Age 17 |

**Supplemental Table 2. Descriptive characteristics of 10,734 adolescents by mental ill-health and overweight or obesity comorbidity status that attended the ages 14- and 17-years assessments of the Millennium Cohort Study. All Values are %**

| | Complete cases N=1,977* | | | 80 Multiply imputed datasets N=11,858 *Sample used in analysis* | | |
|---|---|---|---|---|---|---|
| | **%** | **95% CI** | | **%** | **95% CI** | |
| **Overweight/Obese at age 14 years** | 20.5% | 18.5% | 22.5% | 25.0% | 24.0% | 26.0% |
| **Overweight/Obese at age 17 years**** | 23.2% | 21.1% | 25.4% | 30.1% | 29.1% | 31.0% |
| **Psychological distress at 14 years** | 13.6% | 12.0% | 15.2% | 15.9% | 15.0% | 16.8% |
| **Psychological distress at 17 years (Kessler)**** | 11.5% | 10.1% | 13.1% | 16.2% | 15.5% | 17.0% |
| **Psychological distress at 17 years (SDQ)**** | 20.4% | 18.4% | 22.5% | 22.2% | 21.4% | 23.0% |
| ***Mental ill-heath and overweight or obesity*** | | | | | | |
| Age 14 | 3.6% | 2.7% | 4.7% | 4.9% | 4.4% | 5.3% |
| Age 17 (SDQ emotional subscale)** | 5.2% | 4.2% | 6.3% | 6.8% | 6.1% | 7.4% |
| Age 17 (Kessler Psychological Distress Scale)** | 3.2% | 2.5% | 4.1% | 4.7% | 4.1% | 5.3% |
| ***Cumulative ACE score*** | | | | | | |
| *0 ACEs* | 62.6% | 60.0% | 65.2% | 44.3% | 42.7% | 46.0% |
| *1 ACE* | 27.2% | 25.1% | 29.5% | 29.4% | 28.2% | 30.7% |
| *2 ACEs* | 8.1% | 6.8% | 9.6% | 14.7% | 13.8% | 15.7% |
| *≥3 ACEs* | 2.0% | 1.4% | 2.9% | 11.5% | 10.4% | 12.6% |
| ***Individual ACEs*** | | | | | | |
| **Regular maternal drug use** | 0.4% | 0.2% | 0.9% | 1.6% | 1.2% | 1.9% |
| **Maternal problem drinking behaviour** | 5.2% | 4.2% | 6.5% | 9.1% | 8.1% | 10.2% |
| **Maternal psychological distress** | 8.2% | 6.9% | 9.8% | 25.8% | 24.0% | 27.6% |
| **Bullying** | 8.3% | 7.0% | 9.7% | 14.8% | 13.8% | 15.8% |
| **Physical punishment** | 20.5% | 18.3% | 22.9% | 28.5% | 27.1% | 29.8% |
| **Intimate partner violence** | 6.2% | 5.2% | 7.5% | 18.5% | 17.1% | 19.9% |
| ***Covariates*** | | | | | | |
| **Equivalised household income** | | | | | | |
| Bottom quintile | 2.7% | 2.0% | 3.5% | 16.3% | 15.0% | 17.6% |
| 40% | 8.9% | 7.3% | 10.7% | 17.8% | 16.6% | 19.1% |
| 60% | 21.9% | 19.5% | 24.5% | 20.6% | 19.3% | 21.9% |
| 80% | 28.9% | 26.4% | 31.5% | 21.7% | 20.5% | 22.8% |
| Highest quintile | 37.7% | 33.8% | 41.7% | 23.6% | 21.2% | 26.0% |
| **Sex at birth** | | | | | | |
| Female | 50.0% | 47.4% | 52.6% | 49.7% | 48.6% | 50.8% |
| **Ethnicity** | | | | | | |
| White | 96.5% | 95.0% | 97.5% | 87.4% | 85.3% | 89.5% |
| Mixed ethnicity | 2.1% | 1.4% | 3.2% | 3.0% | 2.5% | 3.5% |
| South Asian | 0.8% | 0.5% | 1.3% | 6.3% | 4.7% | 7.8% |
| Black | 0.3% | 0.2% | 0.7% | 2.1% | 1.4% | 2.8% |
| Other ethnic background | 0.3% | 0.1% | 0.7% | 1.2% | 0.8% | 1.6% |
| **Sexual identity** | | | | | | |
| Sexual minority | 23.3% | 21.3% | 25.5% | 22.1% | 21.0% | 23.3% |

*Those individuals with data all six ACEs and all covariates

**Estimates on age 14 outcomes and ACEs based on N=11,858, estimates on age 17 outcomes based on N=10,950

**Supplemental Table 3. Associations between adverse childhood experiences (ACEs) and mental ill-health and overweight or obesity comorbidity in 10,734 adolescents aged 14 years from the Millennium Cohort Study. Estimates are from multivariable logistic regression models.**

| | **Odds for mental ill-health and overweight or obesity comorbidity** | | | | | | | |
|---|---|---|---|---|---|---|---|---|
| | **Model 1 (unadjusted)[1]** | | | | **Model 2 (adjusted[2])[1]** | | | |
| ***Individual ACEs*** | **OR** | **95% CI** | **p-value** | ***p for trend*** | **OR** | **95% CI** | **p-value** | ***p for trend*** |
| **Maternal drug use** | 1.33 | 0.68-2.56 | 0.40 | | 1.21 | 0.61-2.41 | 0.57 | |
| **Maternal problem drinking behaviour** | 1.34 | 0.71-1.47 | 0.90 | | 1.29 | 0.66-1.39 | 0.81 | |
| **Maternal psychological distress** | **1.66** | **1.33-2.08** | **<0.001** | | **1.51** | **1.15-1.99** | **0.004** | |
| **Intimate partner violence** | **1.35** | **1.10-1.70** | **0.003** | | **1.19** | **1.05-1.56** | **0.03** | |
| **Bullying** | **2.01** | **1.66-2.48** | **<0.001** | | **1.99** | **1.60-2.47** | **<0.001** | |
| **Physical punishment** | 1.16 | 0.95-1.42 | 0.16 | | **1.26** | **1.01-1.55** | **0.037** | |
| ***Cumulative ACE score*** | | | | | | | | |
| **0** | Ref | | | | Ref | | | |
| **1** | **1.22** | **1.01-1.55** | **0.04** | 0.008 | **1.24** | **1.02-1.59** | **0.04** | 0.007 |
| **2** | **1.74** | **1.31-2.29** | **<0.001** | | **1.73** | **1.28-2.34** | **<0.001** | |
| **≥3** | **2.00** | **1.48-2.62** | **<0.001** | | **1.94** | **1.36-2.75** | **<0.001** | |

1. Mental health based on Strengths and Difficulties Questionnaire (SDQ-S) emotional symptoms subscale.
2. Adjusted for sex at birth, ethnicity, sexual identity and parental income. Figures in bold indicate OR and corresponding 95% confidence intervals which do not include 1.

**Supplemental Table 4. Associations between adverse childhood experiences (ACEs) and mental ill-health and overweight or obesity comorbidity in 9,336 adolescents aged 17 years from the Millennium Cohort Study. Estimates are from multivariable logistic regression models.**

| | **Odds for mental ill-health and overweight or obesity comorbidity** | | | | | | | |
|---|---|---|---|---|---|---|---|---|
| | **Model 1 (unadjusted)[1]** | | | | **Model 2 (adjusted[3])[1]** | | | |
| ***Individual ACEs*** | **OR** | **95% CI** | **p-value** | **p-value for trend** | **OR** | **95% CI** | **p-value** | **p-value for trend** |
| **Maternal drug use** | 0.65 | 0.17-2.52 | 0.53 | | 0.54 | 0.13-2.15 | 0.379 | |
| **Maternal problem drinking behaviour** | 1.01 | 0.60-1.69 | 0.972 | | 0.93 | 0.54-1.60 | 0.789 | |
| **Maternal psychological distress** | **1.98** | **1.47-2.68** | **<0.001** | | **1.90** | **1.32-2.75** | **0.001** | |
| **Intimate partner violence** | 1.37 | 0.94-1.99 | 0.101 | | 1.20 | 0.79-1.82 | 0.394 | |
| **Bullying** | **2.21** | **1.68-2.90** | **<0.001** | | **2.14** | **1.58-2.91** | **<0.001** | |
| **Physical punishment** | 0.93 | 0.73-1.19 | 0.563 | | 1.02 | 0.79-1.32 | 0.875 | |
| ***Cumulative ACE score*** | | | | | | | | |
| **0** | Ref | | | 0.012 | Ref | | | 0.013 |
| **1** | 1.30 | 0.93-1.81 | 0.126 | | 1.34 | 0.96-1.88 | 0.087 | |
| **2** | **1.85** | **1.28-2.67** | **0.001** | | **1.89** | **1.27-2.81** | **0.002** | |
| **≥3** | **2.04** | **1.38-3.00** | **<0.001** | | **2.05** | **1.29-3.25** | **0.003** | |
| | **Model 1 (unadjusted)[2]** | | | | **Model 2 (adjusted[3])[2]** | | | |
| ***Individual ACEs*** | **OR** | **95% CI** | **p-value** | **p-value for trend** | **OR** | **95% CI** | **p-value** | **p-value for trend** |
| **Maternal drug use** | 1.79 | 0.89-3.62 | 0.103 | | 1.69 | 0.80-3.56 | 0.168 | |
| **Maternal problem drinking behaviour** | 1.09 | 0.70-1.71 | 0.704 | | 1.05 | 0.65-1.68 | 0.844 | |
| **Maternal psychological distress** | **1.53** | **1.16-2.03** | **0.003** | | **1.50** | **1.05-2.13** | **0.025** | |
| **Intimate partner violence** | 1.15 | 0.85-1.58 | 0.363 | | 1.06 | 0.75-1.50 | 0.738 | |
| **Bullying** | **1.60** | **1.26-2.05** | **<0.001** | | **1.59** | **1.22-2.08** | **0.001** | |
| **Physical punishment** | **0.79** | **0.64-0.99** | **0.040** | | 0.91 | 0.71-1.15 | 0.426 | |
| ***Cumulative ACE score*** | | | | | | | | |
| **0** | Ref | | | | Ref | | | |
| **1** | 1.15 | 0.89-1.50 | 0.289 | 0.201 | 1.20 | 0.92-1.58 | 0.173 | 0.215 |
| **2** | 1.29 | 0.92-1.80 | 0.136 | | 1.33 | 0.92-1.92 | 0.123 | |
| **≥3** | **1.47** | **1.05-2.06** | **0.025** | | **1.54** | **1.04-2.29** | **0.032** | |

1. Mental health based on the 6-item Kessler Psychological Distress Scale (K6) 2. Mental health based on SDQ emotional symptoms subscale. 3. Adjusted for sex at birth, ethnicity, sexual identity and parental income. Figures in bold indicate OR and corresponding 95% confidence intervals which do not include 1.

**Supplemental Table 5. Results from sensitivity analysis. Associations between adverse childhood experiences (ACEs) and mental ill-health and overweight or obesity comorbidity in 9,336 adolescents at ages 14 and 17 years from the Millennium Cohort Study. Estimates are from multivariable logistic regression models.**

| | **Odds for mental ill-health and overweight or obesity comorbidity** | | | | | |
|---|---|---|---|---|---|---|
| **Age 14** | **Model 1 (unadjusted)** | | | **Model 2 (adjusted)[3]** | | |
| | **OR** | **95% CI** | **p-value** | **OR** | **95% CI** | **p-value** |
| Cumulative ACE score | | | | | | |
| 0 | Ref. | | | Ref. | | |
| 1 | 1.17 | 0.87-1.57 | 0.291 | 1.23 | 0.90-1.66 | 0.19 |
| 2 | **1.73** | **1.22-2.45** | **0.002** | **1.74** | **1.18-2.55** | **0.005** |
| | | | | | | |
| **Age 17[1]** | **Model 1 (unadjusted)** | | | **Model 2 (adjusted)[3]** | | |
| | **OR** | **95% CI** | **p-value** | **OR** | **95% CI** | **p-value** |
| Cumulative ACE score | | | | | | |
| 0 | Ref. | | | | | |
| 1 | 0.92 | 0.71-1.19 | 0.54 | 0.99 | 0.75-1.29 | 0.92 |
| 2 | 1.05 | 0.75-1.46 | 0.79 | 1.06 | 0.73-1.53 | 0.76 |
| **Age 17[2]** | **Model 1 (unadjusted)** | | | **Model 2 (adjusted)[3]** | | |
| | **OR** | **95% CI** | **p-value** | **OR** | **95% CI** | **p-value** |
| Cumulative ACE score | | | | | | |
| 0 | Ref. | | | | | |
| 1 | 1.00 | 0.74-1.35 | 0.99 | 1.04 | 0.76-1.43 | 0.80 |
| 2 | 1.20 | 0.83-1.73 | 0.32 | 1.13 | 0.75-1.71 | 0.56 |

1.Mental health based on SDQ emotional symptoms subscale. 2. Mental health based on the 6-item Kessler Psychological Distress Scale (K6). 3. Adjusted for sex at birth, ethnicity, sexual identity and parental income. Figures in bold indicate OR and corresponding 95% confidence intervals which do not include 1.

**Supplemental Table 6. Associations between adverse childhood experiences (ACEs) and mental ill-health and overweight or obesity comorbidity in 10,734 adolescents at age 14 years from the Millennium Cohort Study. Estimates are from multivariable logistic regression models including interaction terms between ethnicity, parental income and sexual identity and cumulative ACE scores**

| | **Odds for mental ill-health and overweight or obesity comorbidity[1]** | | | | | | | | | | | |
|---|---|---|---|---|---|---|---|---|---|---|---|---|
| | **Ethnicity** | | | | **Parental income** | | | | **Sexual identity** | | | |
| | **OR** | **95% CI** | | **p-value** | **OR** | **95% CI** | | **p-value** | **OR** | **95% CI** | | **p-value** |
| ***Cumulative ACE score*** | | | | 0.512[2] | | | | 0.171[2] | | | | 0.98[2] |
| **1** | 1.10 | 0.45 | 2.68 | 0.84 | 1.02 | 0.84 | 1.23 | 0.863 | 0.95 | 0.55 | 1.64 | 0.859 |
| **2** | 0.70 | 0.25 | 1.96 | 0.497 | 1.06 | 0.86 | 1.30 | 0.599 | 1.06 | 0.59 | 1.90 | 0.853 |
| **≥3** | 0.70 | 0.29 | 1.67 | 0.422 | **1.26** | **1.00** | **1.60** | **0.05** | 0.92 | 0.51 | 1.68 | 0.795 |

1. Models adjusted for ACE score, sex at birth, ethnicity, income, sexual identity, but interactions with only ethnicity, income or sexual identity. 2. Overall p-value calculated from Wald tests to judge the overall statistical significance between the number of ACEs and ethnicity/household income/sexual identity.

**Supplemental Table 7. Associations between adverse childhood experiences (ACEs) and mental ill-health and overweight or obesity comorbidity in 9,336 adolescents at age 17 years from the Millennium Cohort Study. Estimates are from multivariable logistic regression models including interaction terms between ethnicity, parental income and sexual identity and cumulative ACE scores**

| Odds for mental ill-health and overweight or obesity comorbidity[1] | | | | | | | | | | | | |
|---|---|---|---|---|---|---|---|---|---|---|---|---|
| | Ethnicity | | | | Parental income | | | | Sexual identity | | | |
| | **OR** | **95% CI** | | **p-value** | **OR** | **95% CI** | | **p-value** | **OR** | **95% CI** | | **p-value** |
| ***Cumulative ACE score[2]*** | | | | 0.707[4] | | | | 0.359[4] | | | | 0.489[4] |
| 1 | 1.04 | 0.32 | 3.38 | 0.953 | 1.08 | 0.87 | 1.35 | 0.496 | 0.73 | 0.41 | 1.30 | 0.286 |
| 2 | 1.36 | 0.39 | 4.71 | 0.624 | 1.10 | 0.85 | 1.42 | 0.469 | 0.87 | 0.44 | 1.72 | 0.678 |
| ≥3 | 0.86 | 0.29 | 2.57 | 0.781 | 1.07 | 0.80 | 1.44 | 0.644 | 0.64 | 0.32 | 1.29 | 0.209 |
| | | | | | | | | | | | | |
| ***Cumulative ACE score[3]*** | | | | 0.617[4] | | | | 0.157[4] | | | | 0.299[4] |
| 1 | 1.40 | 0.38 | 5.19 | 0.608 | 1.01 | 0.94 | 0.79 | 0.08 | 0.75 | 0.39 | 1.43 | 0.38 |
| 2 | 1.58 | 0.40 | 6.18 | 0.512 | 1.05 | 0.73 | 0.79 | 0.35 | 0.94 | 0.45 | 1.98 | 0.87 |
| ≥3 | 0.97 | 0.29 | 3.29 | 0.96 | 1.23 | 0.21 | 0.89 | 1.24 | 0.69 | 0.31 | 1.54 | 0.37 |

1. Models adjusted for ACE score, sex at birth, ethnicity, income, sexual identity, but interactions with only ethnicity, income or sexual identity 2. Mental health based on SDQ emotional symptoms subscale. 3. Mental health based on the 6-item Kessler Psychological Distress Scale (K6). 4. Overall p-value calculated from Wald tests to judge the overall statistical significance between the number of ACEs and ethnicity/household income/sexual identity.
